# Supplementary material for: Chemoinformatic Screening for the Selection of Potential Senolytic Compounds from Natural Products
Source: Biomolecules. 2021 Mar 22;11(3):467. doi: 10.3390/biom11030467 (PMC8004226; doi:10.3390/biom11030467)
Supplement: Supplementary file 1 [file biomolecules-11-00467-s001.zip › supplementary_figures.pdf]

Supplementary figures

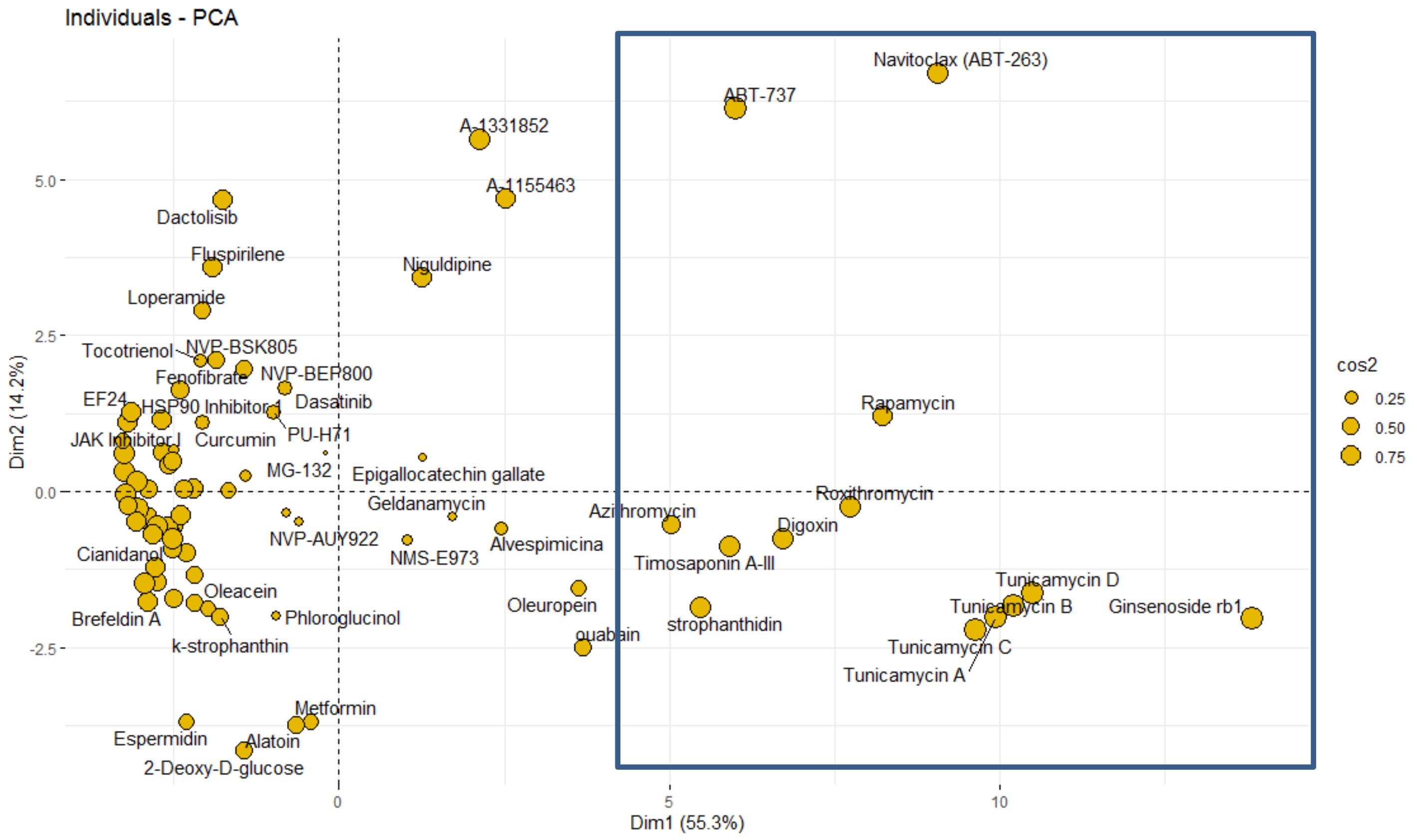

Figure-1. Principal component analysis (PCA) score plot of the analyzed senolytic compounds with respect to their molecular descriptors. GPCR.ligand, Ion.channel.modulator, Kinase.inhibitor, Nuclear.receptor.ligand, Protease.inhibitor, Enzyme.inhibitor, nviolations, natoms, log.Kp..cm.s., Lipinski..violations, Ghose..violations, Veber..violations, Egan..violations, Muegge..violations, Bioavailability.Score, Molweight, cLogP, cLogS, H.Acceptors, H.Donors, Total.Surface.Area, Polar.Surface.Area, Druglikeness, Shape.Index, Molecular.Flexibility, Electronegative.Atoms, Rotatable.Bonds, Aromatic.Rings, Aromatic.Atoms, sp3.Atoms, and Symmetric.atoms.

Navitoclax clustering

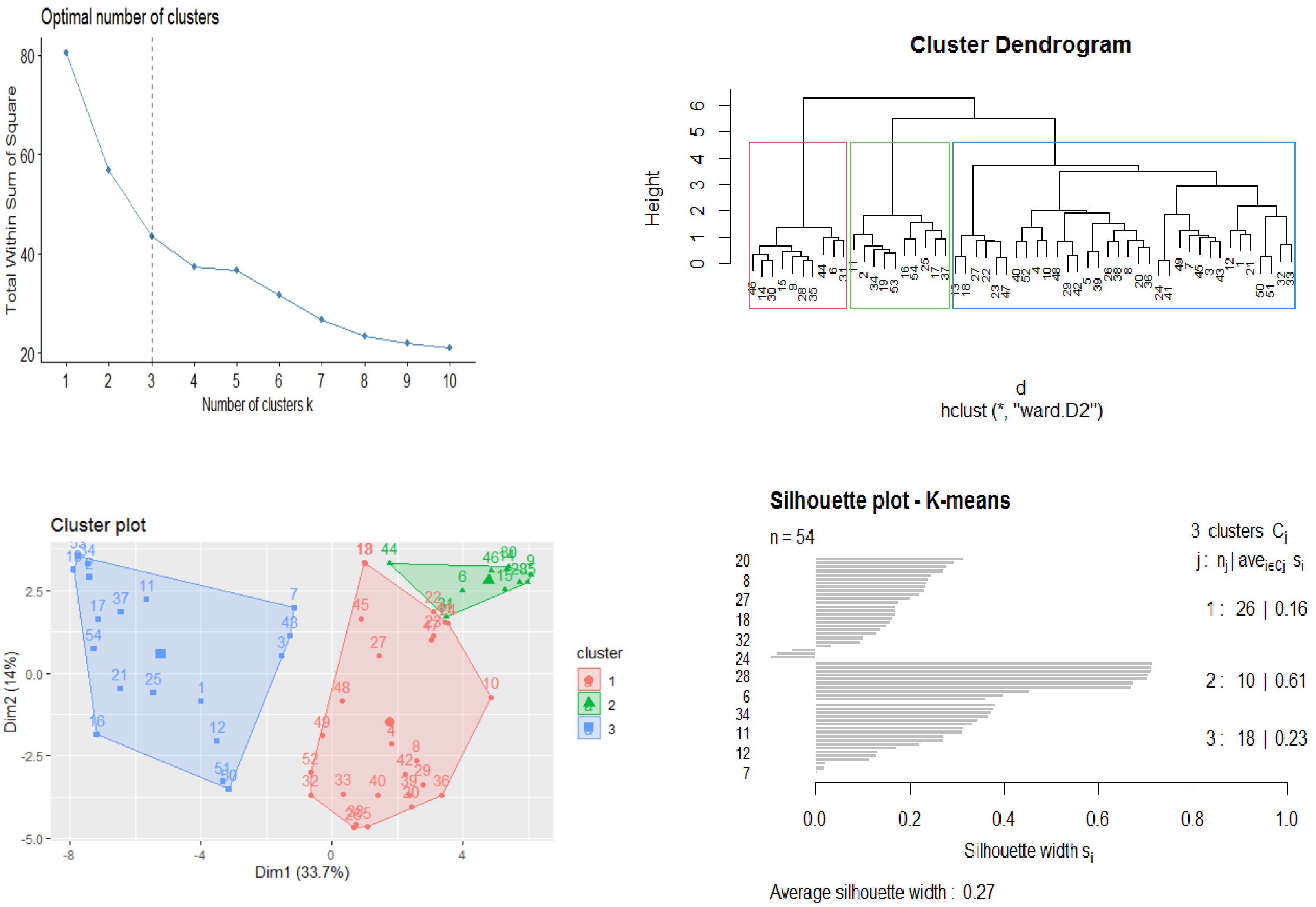

ABT737 clustering

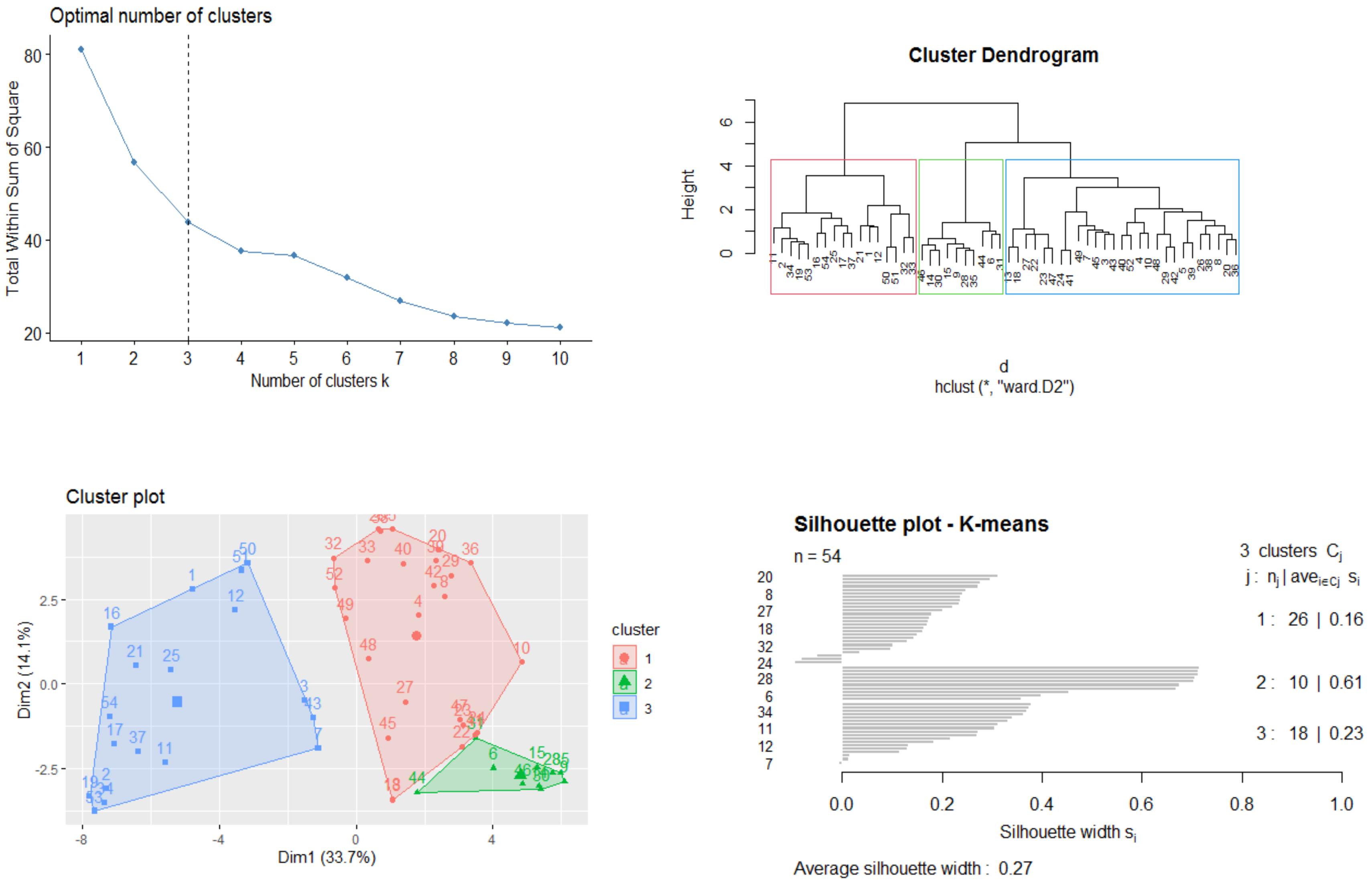

Tunicamycin A clustering

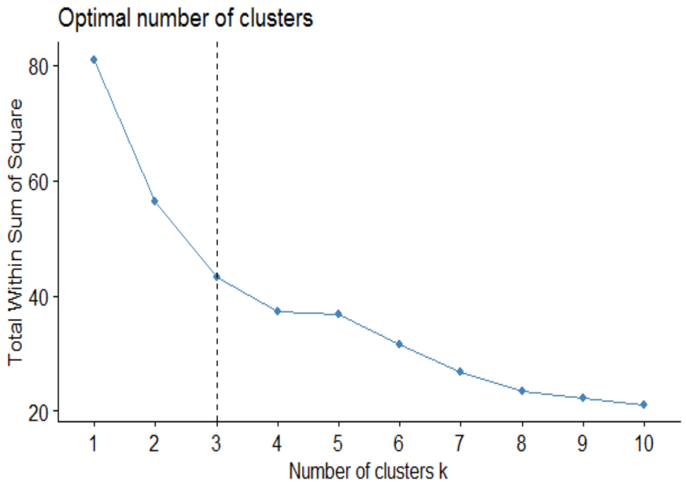

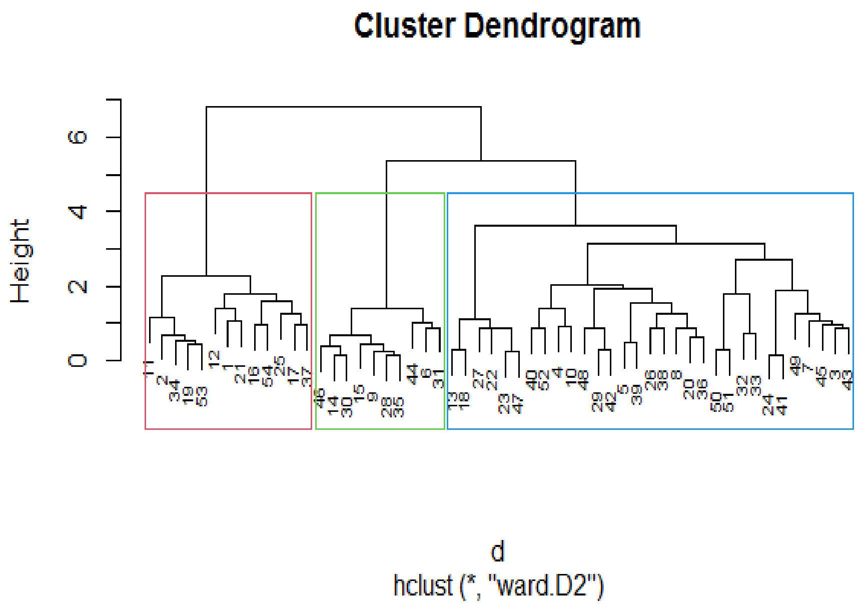

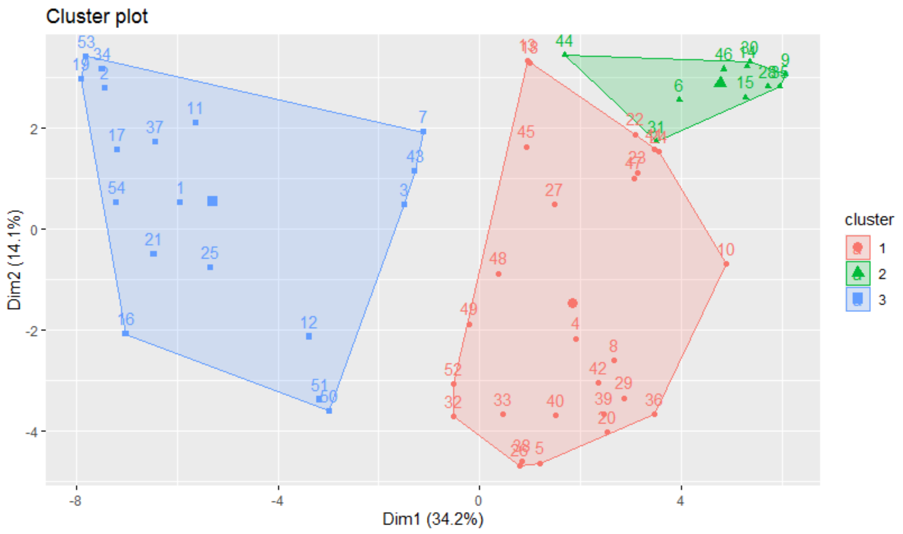

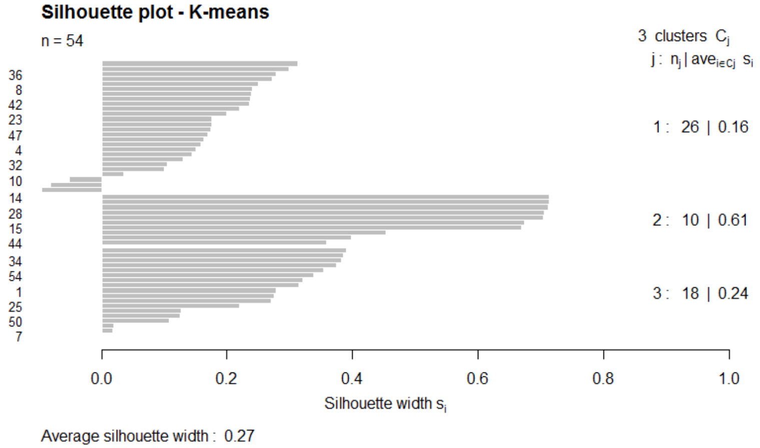

Tunicamycin B clustering

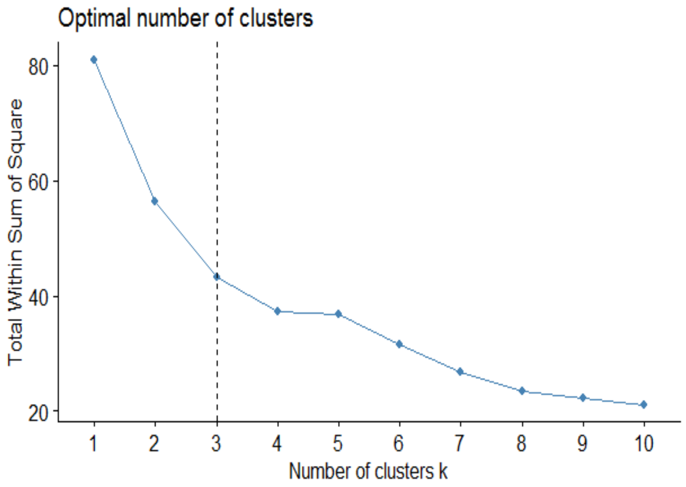

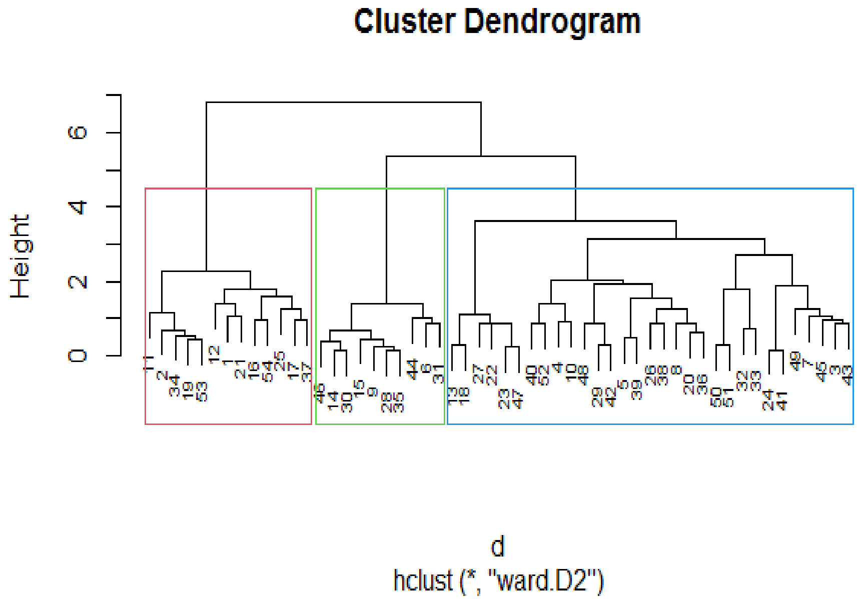

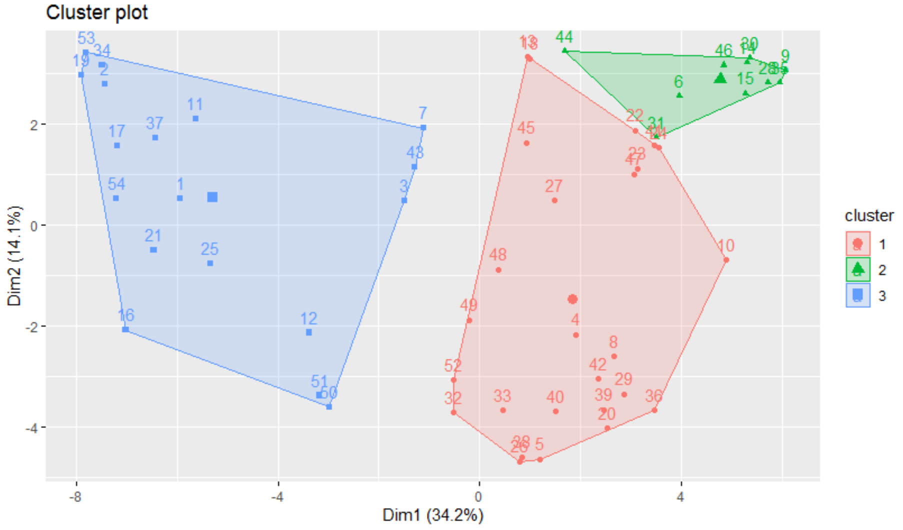

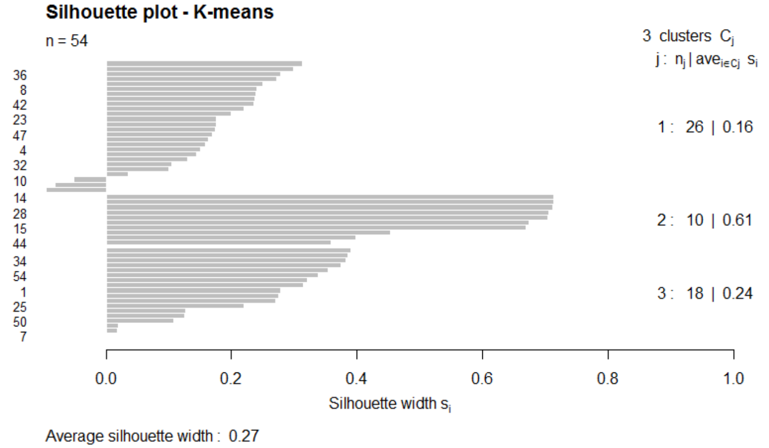

Tunicamycin C clustering

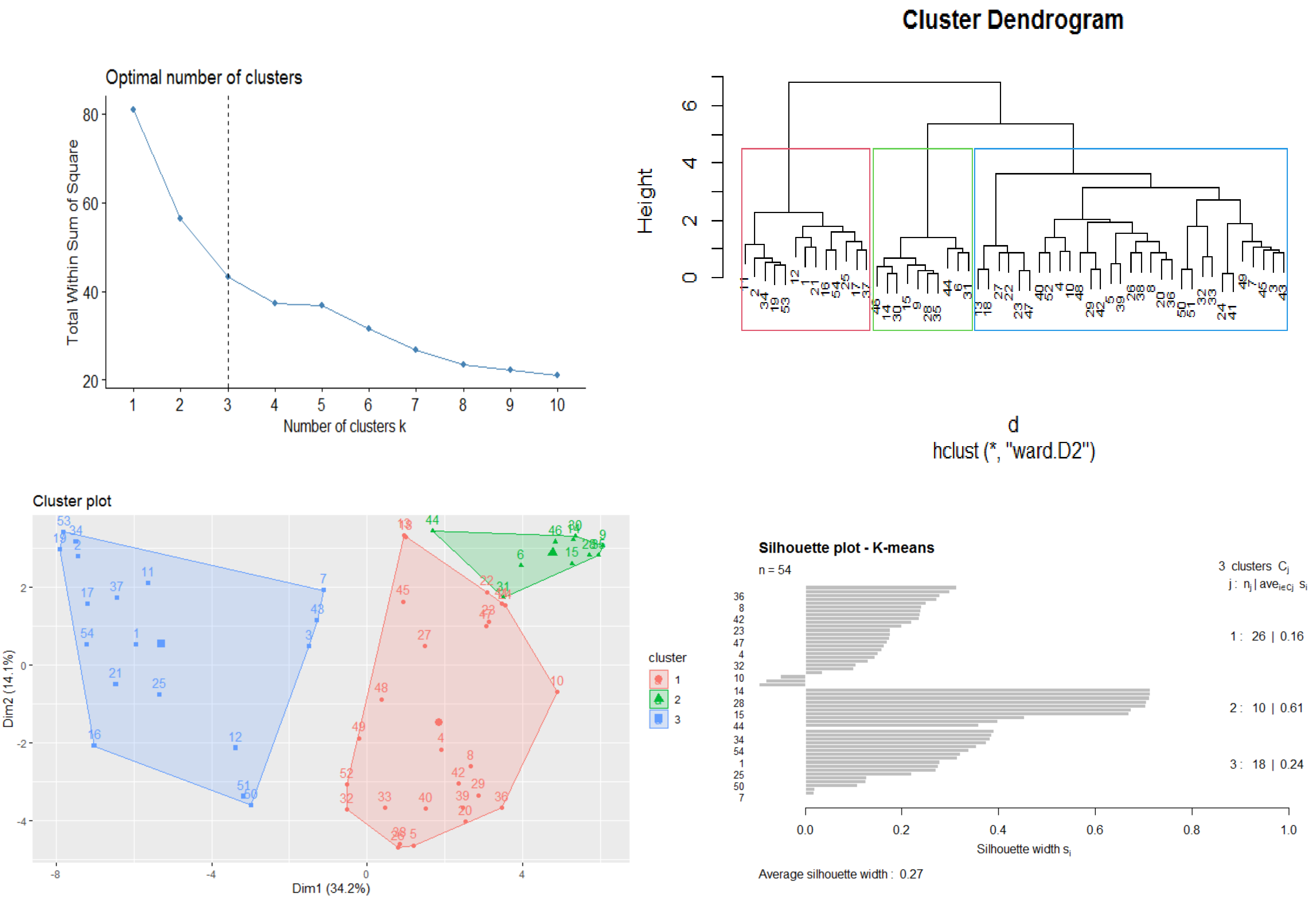

Tunicamycin D clustering

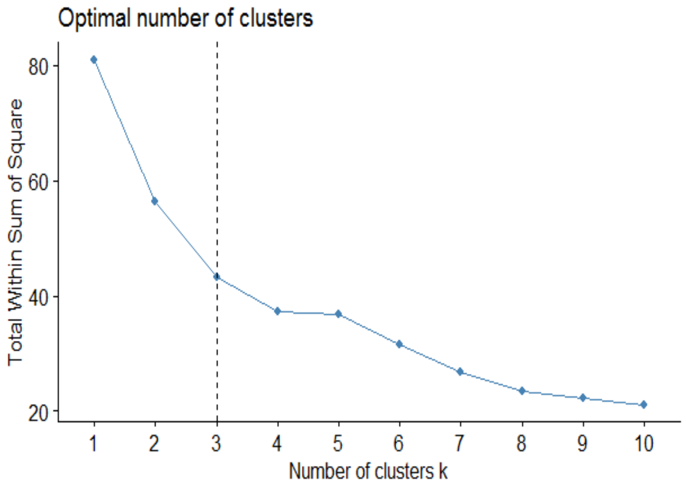

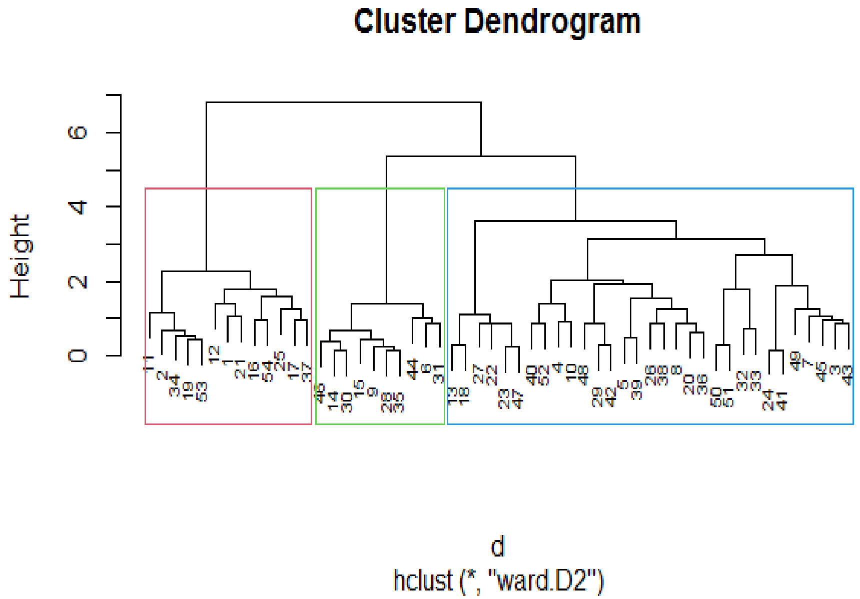

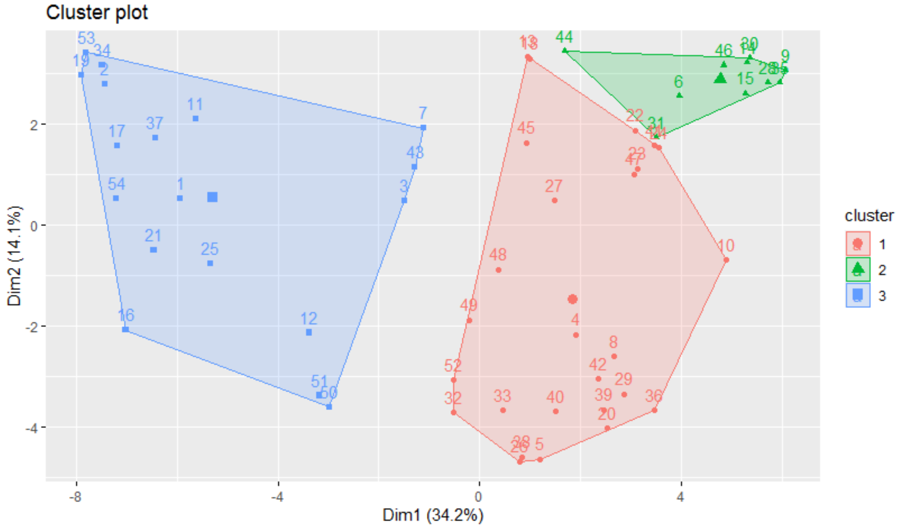

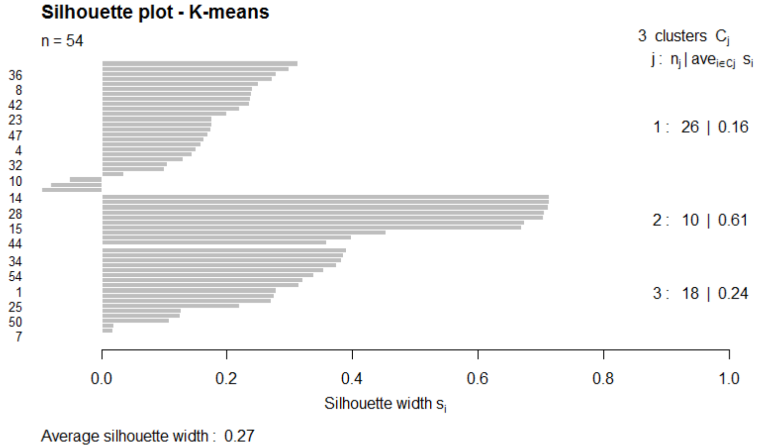

Gingenoside Rb1 clustering

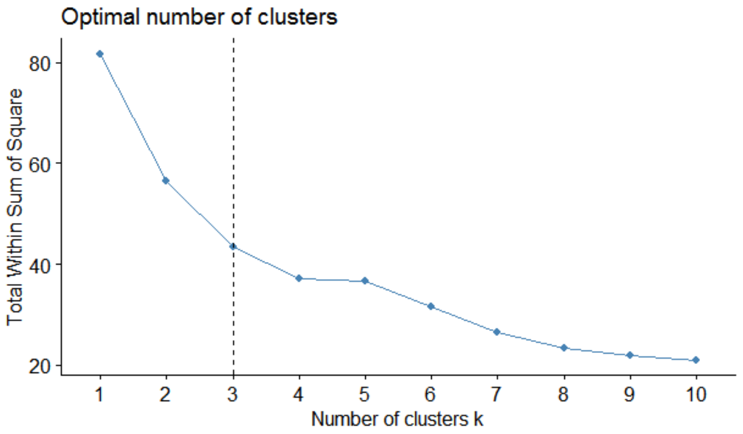

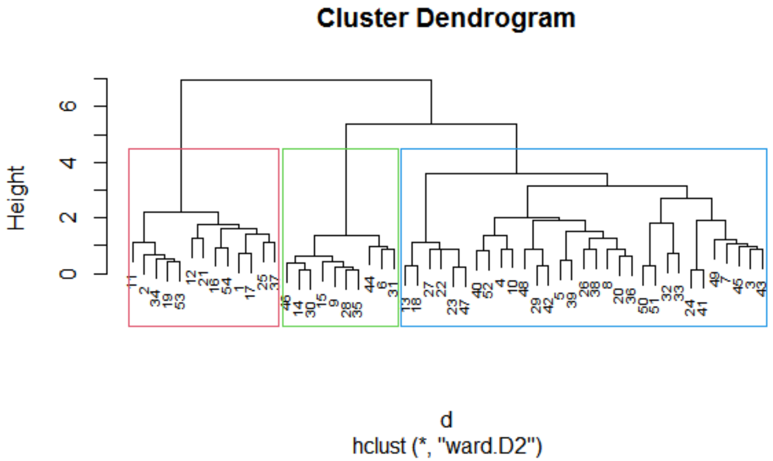

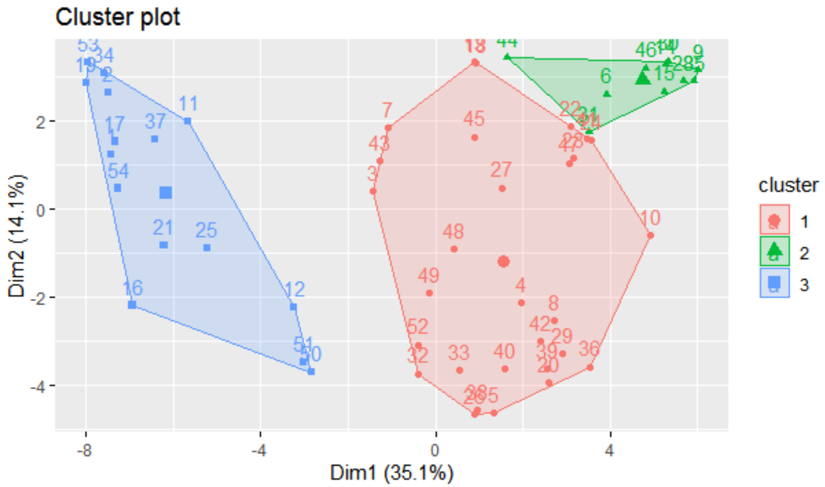

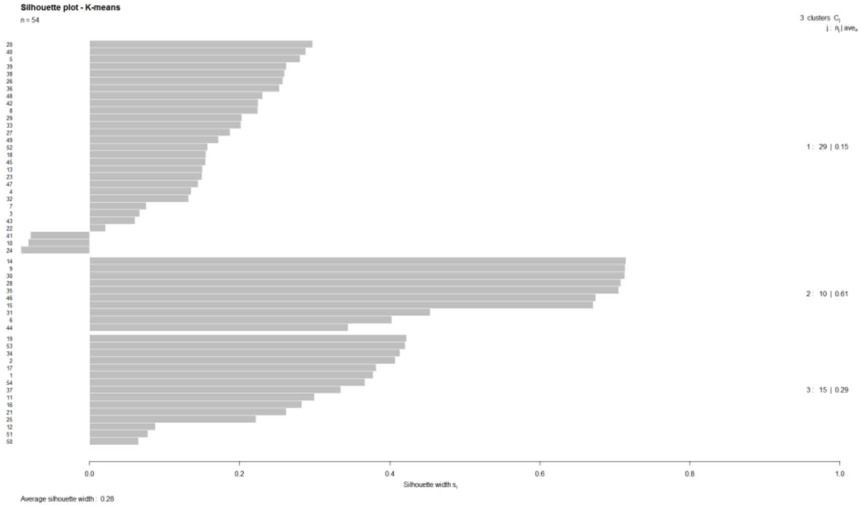

Timosaponin aIII clustering

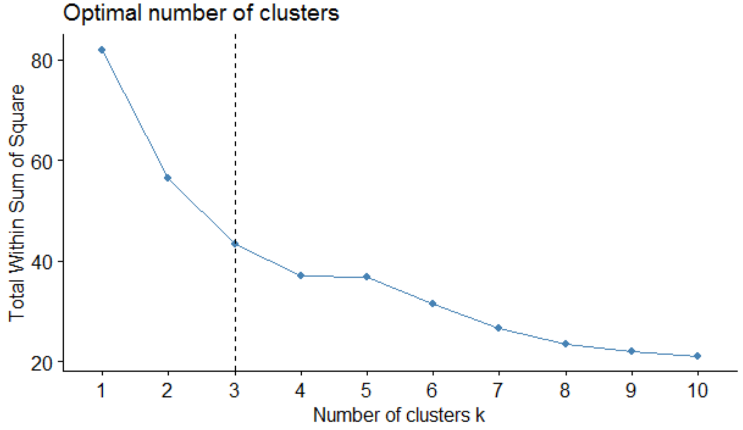

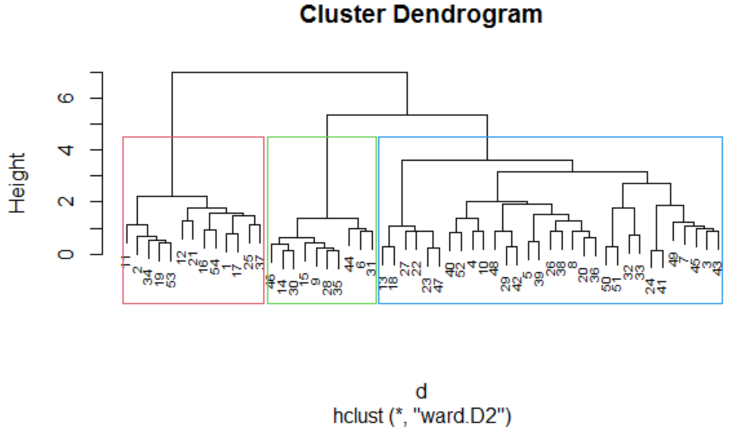

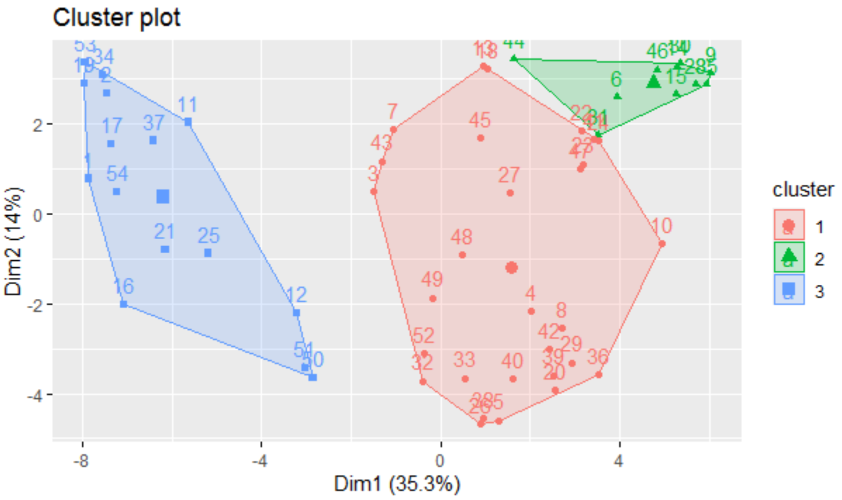

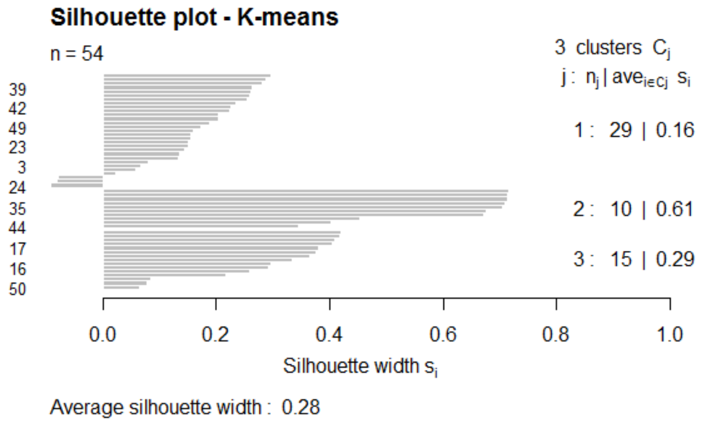

Digoxin clustering

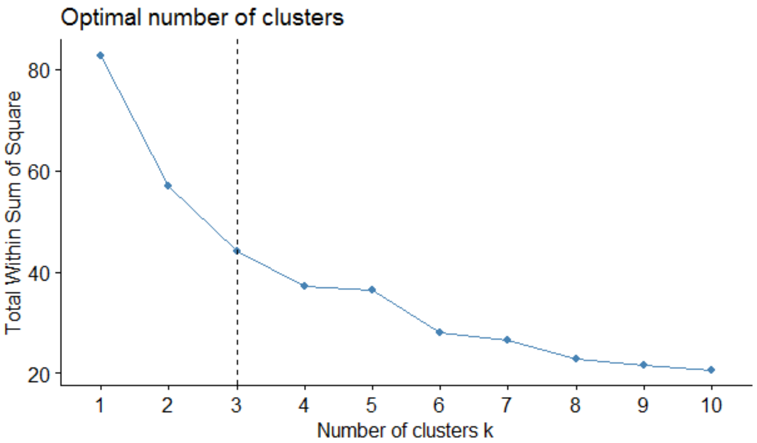

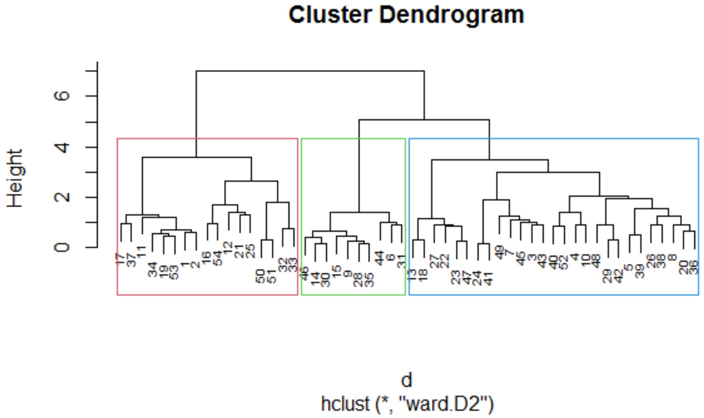

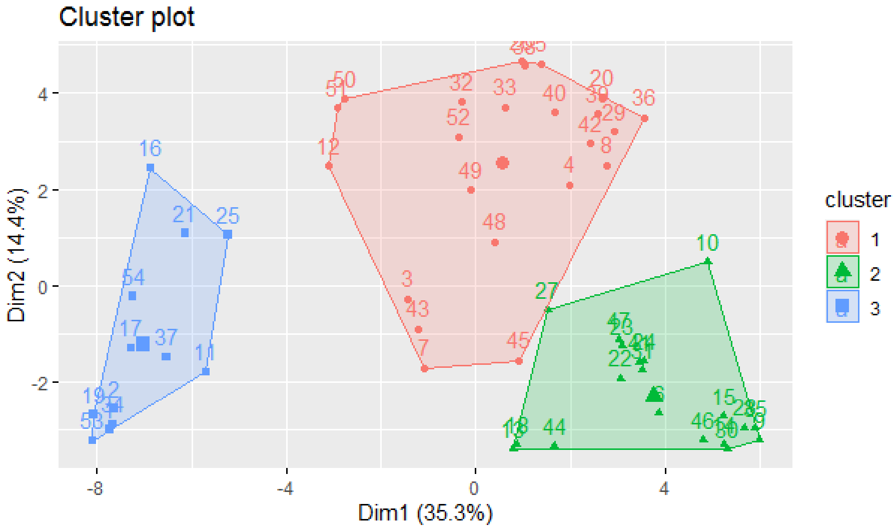

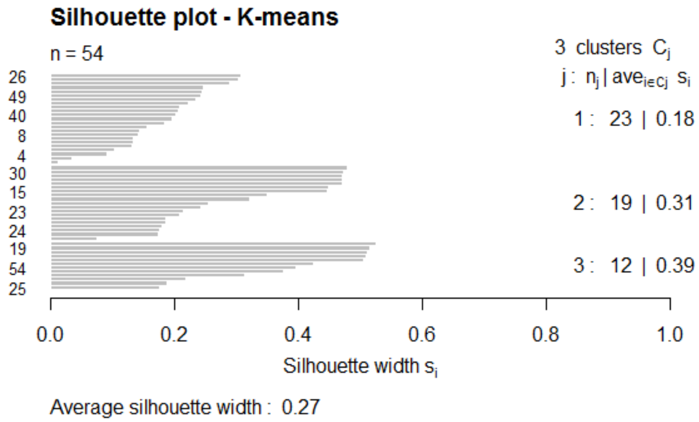

Rapamycin clustering

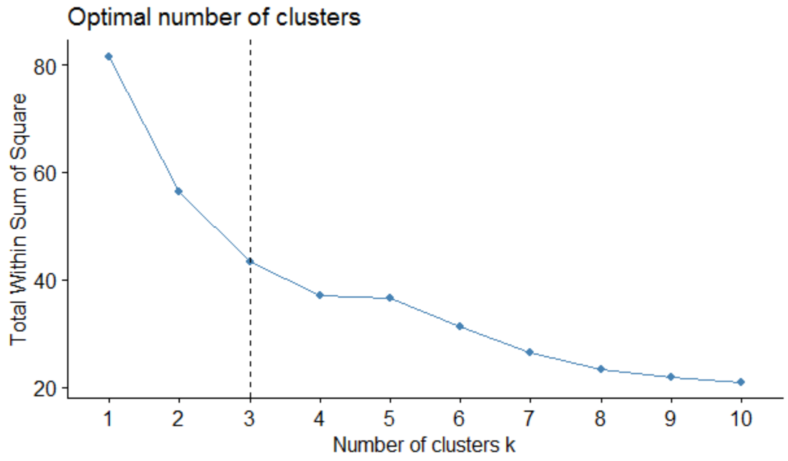

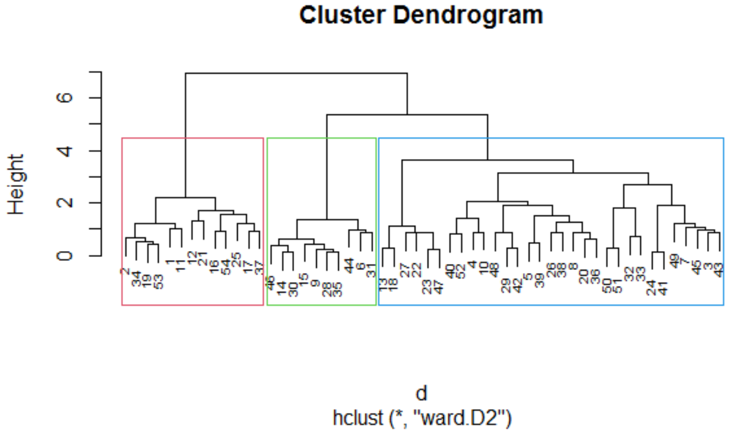

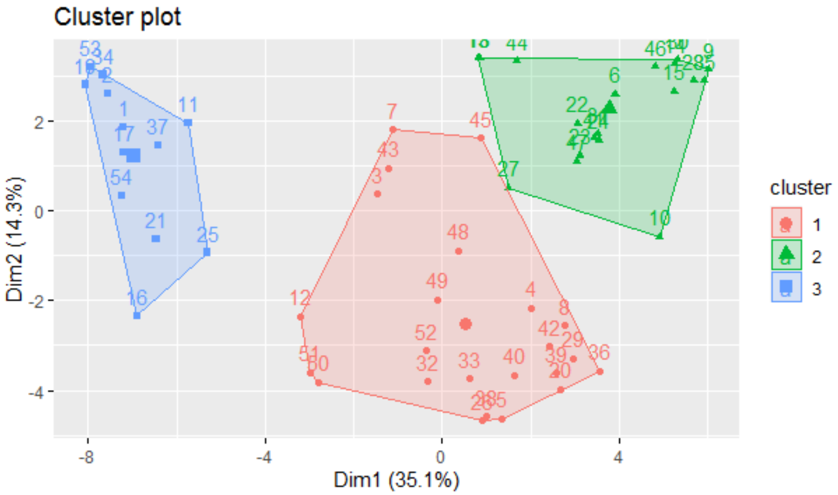

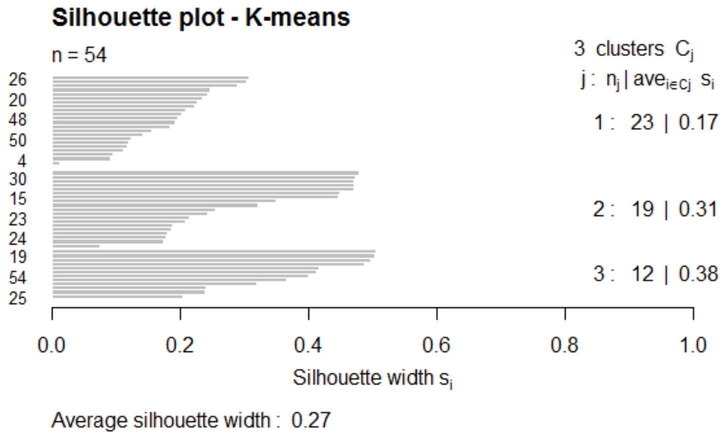

Roxithromycin clustering

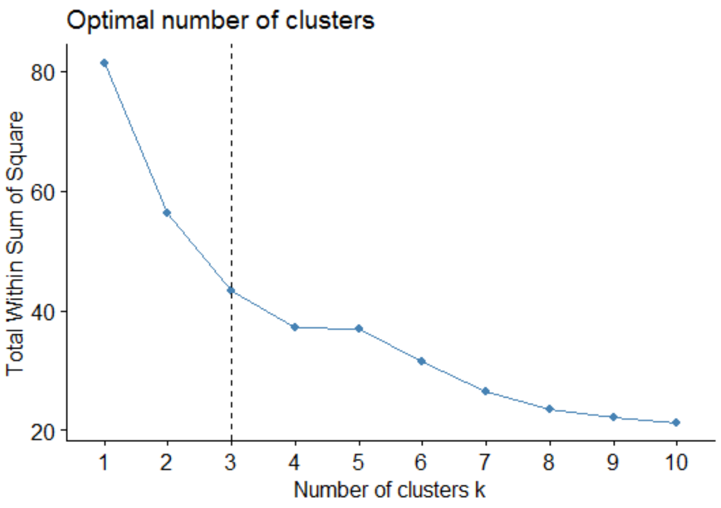

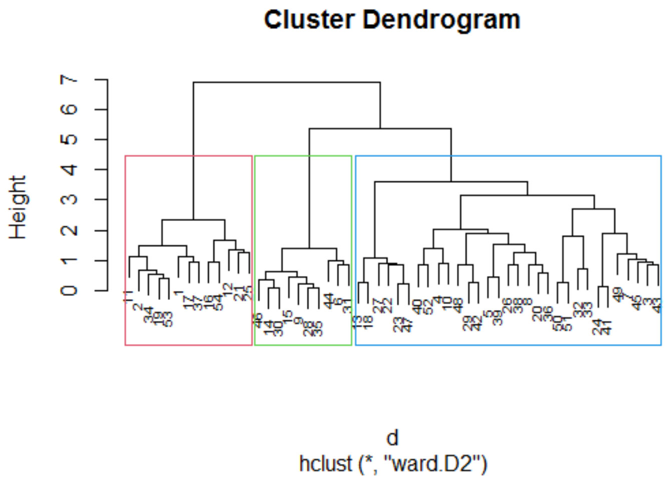

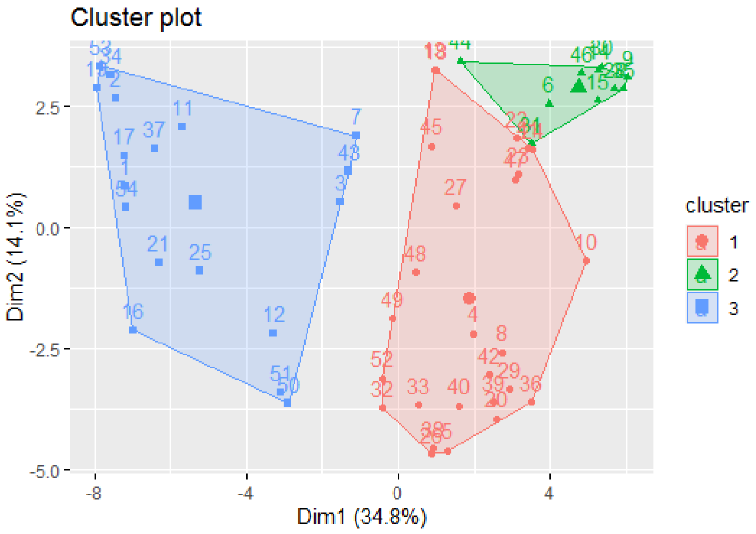

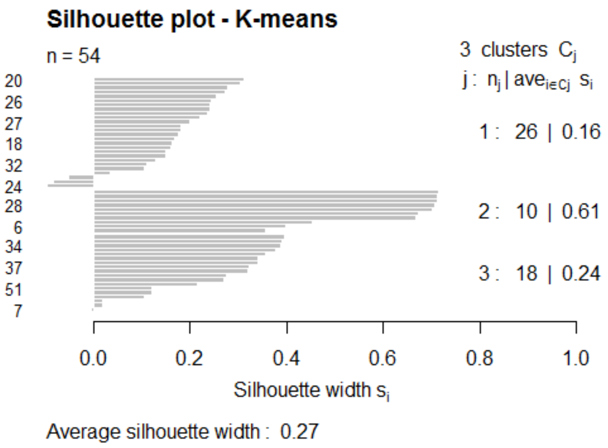

Azithromycin clustering

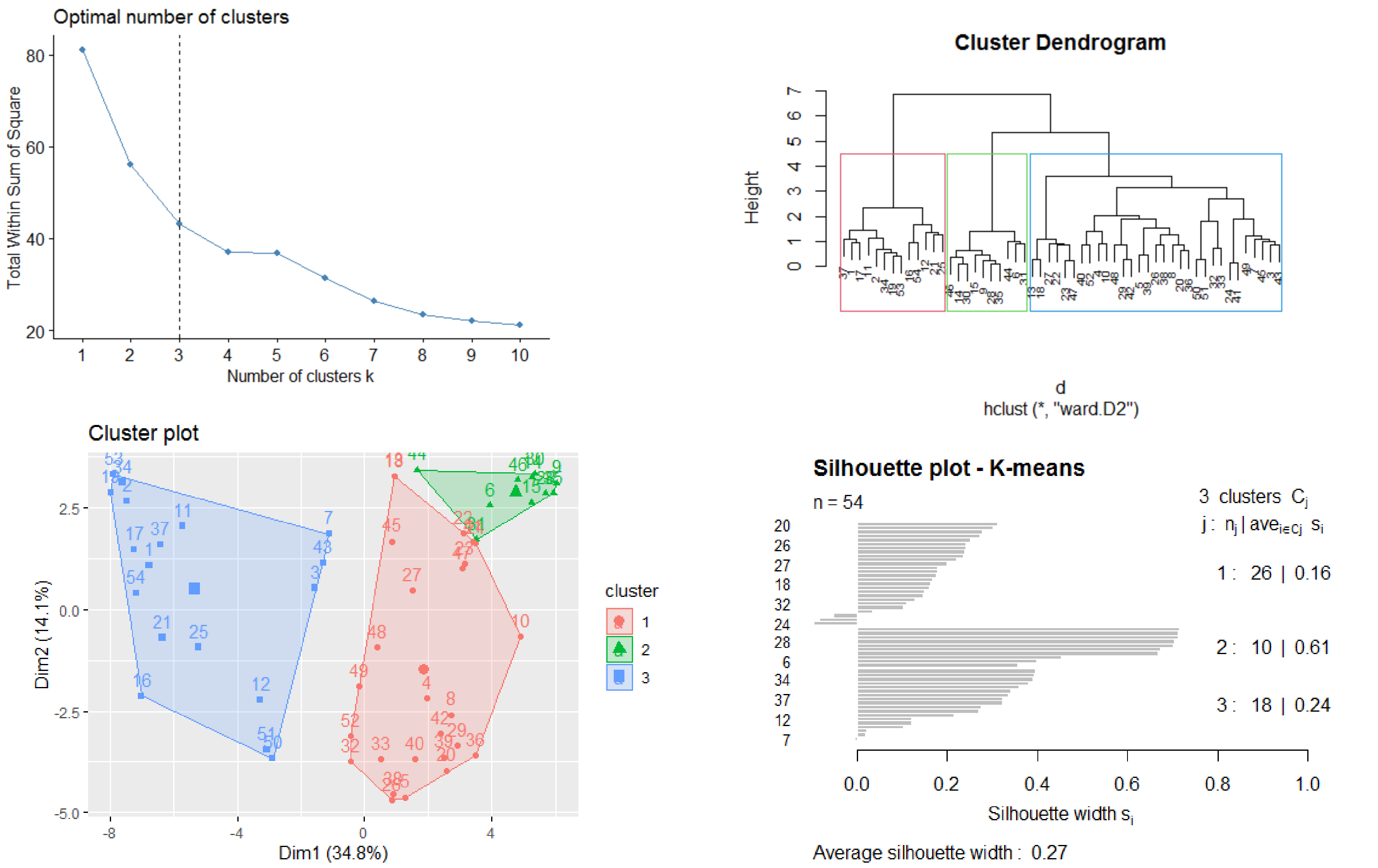

Figure-2. Representative results of the comparison among NPs and senolytic leads by fingerprints. The following steps were carried out for the selection of putative senolyitics employing the comparison among 53 NPs with the senolytic lead (n=54): A) The suitable number of clusters was determined by Elbow method.
B) Clusterization of Ward's method, the senolytic cluster are marked in red and senolytic molecule as 1. C) Cluster plot using Kmeans showing the same molecules in the senolytic cluster marked in blue. D) Silhouette cluster representation to corroborate the first clusterization methods.
